# Interface Symbiotic Membrane Formation in Root Nodules of *Medicago truncatula*: the Role of Synaptotagmins *MtSyt1*, *MtSyt2* and *MtSyt3*

**DOI:** 10.3389/fpls.2017.00201

**Published:** 2017-02-20

**Authors:** Aleksandr Gavrin, Olga Kulikova, Ton Bisseling, Elena E. Fedorova

**Affiliations:** ^1^Laboratory of Molecular Biology, Department of Plant Sciences, Graduate School Experimental Plant Sciences, Wageningen UniversityWageningen, Netherlands; ^2^Sainsbury Laboratory, University of CambridgeCambridge, UK

**Keywords:** symbiosis, synaptotagmin1, membrane tension/repair, interface membrane, root nodule, arbuscular mycorrhiza, *Medicago truncatula*

## Abstract

Symbiotic bacteria (rhizobia) are maintained and conditioned to fix atmospheric nitrogen in infected cells of legume root nodules. Rhizobia are confined to the asymmetrical protrusions of plasma membrane (PM): infection threads (IT), cell wall-free unwalled droplets and symbiosomes. These compartments rapidly increase in surface and volume due to the microsymbiont expansion, and remarkably, the membrane resources of the host cells are targeted to interface membrane quite precisely. We hypothesized that the change in the membrane tension around the expanding microsymbionts creates a vector for membrane traffic toward the symbiotic interface. To test this hypothesis, we selected calcium sensors from the group of synaptotagmins: *MtSyt1, Medicago truncatula* homolog of *AtSYT1* from *Arabidopsis thaliana* known to be involved in membrane repair, and two other homologs expressed in root nodules: *MtSyt2* and *MtSyt3.* Here we show that *MtSyt1, MtSyt2*, and *MtSyt3* are expressed in the expanding cells of the meristem, zone of infection and proximal cell layers of zone of nitrogen fixation (*MtSyt1, MtSyt3)*. All three GFP-tagged proteins delineate the interface membrane of IT and unwalled droplets and create a subcompartments of PM surrounding these structures. The localization of MtSyt1 by EM immunogold labeling has shown the signal on symbiosome membrane and endoplasmic reticulum (ER). To specify the role of synaptotagmins in interface membrane formation, we compared the localization of MtSyt1, MtSyt3 and exocyst subunit EXO70i, involved in the tethering of post-Golgi secretory vesicles and operational in tip growth. The localization of EXO70i in root nodules and arbusculated roots was strictly associated with the tips of IT and the tips of arbuscular fine branches, but the distribution of synaptotagmins on membrane subcompartments was broader and includes lateral parts of IT, the membrane of unwalled droplets as well as the symbiosomes. The double silencing of synaptotagmins caused a delay in rhizobia release and blocks symbiosome maturation confirming the functional role of synaptotagmins. In conclusion: synaptotagmin-dependent membrane fusion along with tip-targeted exocytosis is operational in the formation of symbiotic interface.

## Introduction

The legume-*rhizobium* and plant-arbuscular mycorrhizal symbioses are rare examples in the plant kingdom of intracellular microbes are being tolerated by the host cell for the long periods up to several weeks. Accommodation of microsymbionts causes profound morphological changes of host cell. During the entrance of infection thread to the host cell the division of bacterial cells within a physically confined space provides a “push” mechanism for entry, which is able to counteracting the turgor pressure of the host plant cell (Brewin, 2004). The entrance of microsymbionts triggers the formation of symbiosis-specific asymmetric protrusions of plasma membrane (PM). In legume root nodules such protrusions are enveloping tubular structures called infection threads (IT), infection droplets (unwalled extensions of ITs) and symbiosomes (released bacteria surrounded by a host cell-derived membrane; Roth and Stacey, 1989; Brewin, 2004; Gibson et al., 2008; Kondorosi et al., 2013). In symbiosis with arbuscular mycorrhiza PM protrusions envelop the intracellular branched hyphae called arbuscules (Parniske, 2000; Gutjahr and Parniske, 2013). It is remarkable that the membrane resources of the host cells are targeted to interface membrane surrounding these symbiotic compartments quite precisely in time and space, ensuring, for example, the tip growth of ITs and arbuscules and isodiametric expansion for symbiosomes and unwalled droplets. Till now the mechanisms of such meticulously correct delivery are not known.

The PM is known to be inelastic, unable to stretch more than 3% (Apodaca, 2002). Exocytosis of new membrane material is therefore crucial for the increase in membrane surface area (Grefen et al., 2011). In animal cells, a local increase in membrane tension induces membrane repair mechanisms in that specific region of the membrane (Morris and Homann, 2001). The response of the cells to mechanical stress involves Ca^2+^ spiking, as well as phospholipase signaling, rapid remodeling of the actin skeleton and quick retargeting of membrane resources to reduce the membrane tension (Jaffe et al., 2002; Telewski, 2006). The membrane fusion is achieved by the action of N-ethylmaleimide sensitive factor attachment protein receptors (t-SNAREs), as well as to the vesicle-associated membrane protein (VAMP or v-SNAREs) forming a SNARE complex in a Ca^2+^ dependent process (Adolfsen et al., 2004; Maximov et al., 2009; Falkowski et al., 2011). Local higher concentration of cytoplasmic calcium is required for the targeted vesicular fusion, but the SNARE complex lacks specific Ca^2+^-binding sites. This highlights the crucial role of calcium sensors like synaptotagmins in the process of locally targeted membrane fusion (Reddy et al., 2001; McNeil and Kirchhausen, 2005; Idone et al., 2008; Gauthier et al., 2009). Synaptotagmins are able to bind to t-SNAREs as well as to v-SNAREs hence the association of SNAREs with synaptotagmin may provide Ca^2+^ sensitivity to direct the rapid response that is needed for the fusion of the membrane vesicles with the region of the overstretched (Draeger et al., 2011).

Synaptotagmins have been found in plants (Craxton, 2004; Nakagawa et al., 2007; Schapire et al., 2008; Yamazaki et al., 2008, 2010). In *Arabidopsis thaliana AtSYT1* is involved in membrane repair in the case of osmotic misbalance or cold stress (Schapire et al., 2008; Yamazaki et al., 2008, 2010). Recently is was found that *AtSYT1* is a plant homolog of the mammalian extended synaptotagmins and on the membrane it is enriched on the ER-PM contact sites (Manford et al., 2012; Levy et al., 2015; Pérez-Sancho et al., 2015). AtSYT1 is preferentially localized to ER–PM contact sites also modulating the abundance of PEN1, a component of SNARE membrane fusion complex of PM (Kim et al., 2016).

The expansion of PM in plants depends on targeted exocytosis; the classical examples are the tip growth of root hairs and pollen tubes (Hepler and Winship, 2010; Qin and Dong, 2015) and the formation of the cell plate during cell division (El Kasmi et al., 2013). Tip growth is concomitant with the establishment of a tip-focused Ca^2+^ gradient, as well as with actin microfilament reorganization and redirection of small GTPases of the Rab, Rop and ARF families involved in membrane fusion (Ketelaar et al., 2003; Šamaj et al., 2006). The tethering of post-Golgi vesicles to the fast growing membrane subcompartments on the tip is regulated by the PM protein complex exocyst (He and Guo, 2009; Zárský et al., 2013; Zhang et al., 2015; Vukašinović et al., 2016).

The growth of symbiotic structures in infected cells is anisodiametric for infection threads and arbuscules, and isodiametric for unwalled droplets and symbiosomes. That means that the membrane resources have to be targeted to support the expansion indiscriminately of the type of growth. During endosymbiosis establishment the mechanical pressure over the host PM considered to be a potential signal monitored by plants (Hardham et al., 2008; Chehab et al., 2009; Jayaraman et al., 2014). We hypothesized that the membrane tension caused by expanding microsymbionts creates a spatial vector for membrane fusion to the interface membrane ensuring the enlargement of the symbiotic interface.

To test this hypothesis, we studied *MtSyt1, M. truncatula* homolog of *AtSYT1* shown to be involved in membrane repair (Schapire et al., 2008; Yamazaki et al., 2008) and two other homologs expressed in root nodules: *MtSyt2* and *MtSyt3.* The expression analysis, the localization of GFP-tagged synaptotagmins and functional analysis of the nodules from the roots carrying double silencing constructs shows that synaptotagmins *MtSyt1, MtSyt2*, and *MtSyt3* are co-opted into the process of intracellular accommodation of microsymbionts and involved in the formation of interface membrane. Thereafter the synaptotagmin-dependent membrane fusion and putative membrane repair pathway along with tip-targeted exocytosis is operational in the formation of symbiotic interface.

## Materials and Methods

### Plant Materials, Transformation, and Inoculation

The *Medicago truncatula* accession Jemalong A17 was grown in perlite saturated with Färhaeus medium without nitrate in a growth chamber at 21°C and 16/8-h light/darkness cycle. These plants were inoculated with *Sinorhizobium meliloti* strain Sm2011 (OD600 0.1, 2 mL per plant). Root nodules were collected for analysis 14 days post inoculation (dpi). *Agrobacterium rhizogenes* MSU440-mediated hairy root transformation was performed according to Limpens et al. (2005).

### Cloning

DNA sequences of *M. truncatula* synaptotagmins named *MtSyt1*, *MtSyt2* and *MtSyt3* were retrieved by BLAST based on homology with *Arabidopsis SYT1* (Schapire et al., 2008). *MtSyt1, MtSyt2* and *MtSyt3* genes and their putative promoters were amplified by PCR on *M. truncatula* cDNA genomic DNA, respectively, using Phusion High-Fidelity DNA Polymerase (Finnzymes) and specific primers (Supplementary Table 1). PCR fragments were introduced in pENTR-D-TOPO (Invitrogen) and sequenced. Each promoter was further re-cloned into two different pENTR 4-1 vectors (Invitrogen); one is with GFP and the other without GFP (Invitrogen). To create N- or C- terminal GFP fusion these vectors together with pENTR-TOPO containing *MtSyts* genes and pENTR 2–3 (with or without GFP) were subsequently recombined into the modified pKGW-UBQ10::DsRED destination vector (Limpens et al., 2004) using Gateway technology (Invitrogen), pEnter clone of Exo70i was recombined to the plasmid of UBQ3-pK7WGF2-R (Limpens et al., 2009) creating N-terminal GFP fusion under Ubiquitin promoter (ProUbiq3:GFP-Exo70i).

The double RNAi constructs were created by fusing *MtSyt1*, *MtSyt2* and *MtSyt3* PCR-generated sequences using protocol adopted from Franssen et al. (2015). In a first round of PCR the short overlaps (15 bp) were introduced to PCR products by using specific primers. A mixture of two obtained PCR fragments diluted in 1:500 was used as a template in second PCR to create a single DNA fragment. The primers are listed in Supplementary Table 1.

### Quantitative PCR Analysis

Total RNA was extracted from roots and 14-dpi root nodules using the E.Z.N.A. The extraction was made from two transformations. Plant RNA Mini Kit (Omega Bio-Tek) and transcribed into cDNA using the iScript cDNA synthesis kit (Bio-Rad). Real-time PCR was set up in a 20 μl reaction system using iQ SYBR Green Supermix (Bio-Rad). Gene-specific primers were designed with Primer-3-Plus software (Untergasser et al., 2007). Gene expression profiles were normalized against the transcription level of the reference gene UBQ10. The primers are listed in Supplementary Table 1. Results were compared with *M. truncatula* Gene Expression Atlas^1^ and Symbimics^2^ data. According to Symbimix database (Roux et al., 2014; INRA^2^). MtSyt1, MtSyt2 and MtSyt3 were expressed in the meristem, zone of infection and zone of nitrogen fixation; MtSyt1 and MtSyt3 have higher expression than MtSyt2 (Supplementary Figure 2B).

### GUS Staining

Transgenic roots and nodules were collected and washed twice in 0.1M sodium phosphate buffer, pH 7.2, incubated in β-glucuronidase (GUS) buffer under vacuum at room temperature for 30 min to allow the buffer to replace oxygen in the tissue, incubated at 37°C for 2 h. Hand-cut sections of processed nodules were analyzed using Leica DM 5500 Flu microscopes.

### Confocal Laser-Scanning Microscopy

GFP-fused proteins were visualized on transgenic roots and hand-sectioned nodules. Imaging was done on a Zeiss LSM 5 Meta confocal laser-scanning microscope (Carl Zeiss) and Leica TCS SP8 HyD confocal microscope (Leica) with 63 oil immersion objective. Polyclonal rabbit anti-GFP antibody (Molecular Probes) at a dilution of 1:100 and secondary anti-rabbit Alexa 488 antibody (Molecular Probes), (excitation max 490, emission max 525 nm) at a dilution of 1:200 were used for signal enhancement. The mixture of Goat serum (50%) with 2% (vol/wt) BSA was used as the blocking agent. Sections were counterstained with FM4-64 (30 μg/mL) or propidium iodide (0.001%).

### Sample Preparation for Light and Electron Microscopy (EM)

Tissue preparation was performed as described previously (Limpens et al., 2009). Semi-thin (0.6 μm) sections were cut using a Leica Ultracut microtome (Leica) and examined using a Leica FL light microscope. For EM immunogold analysis the tissue was fixed by high-pressure freezing method as described before by Limpens et al. (2009). The nickel grids with the sections were blocked in normal goat serum with 1% of skimmed milk or 2% BSA in PBS and incubated with the primary antibody at the dilutions given above. Goat anti-rabbit coupled with 10-nm or with 15 nm gold particles (BioCell) (1:50 dilution) were used as secondary antibody. Sections were examined using a JEOL JEM 2100 transmission electron microscope equipped with a Gatan US4000 4K × 4K camera.

### Western Blot Analysis

The proteins were extracted from root nodules in 0.025M Tris-HCl buffer containing 1 mM EDTA, 1 mM DTT and protease inhibitors cocktail (Roche). The probes loaded to the gel: 45 μg/well of root tips extracts of transgenic roots containing GFP-tagged synaptotagmins. The proteins were separated by 12.5% SDS-PAGE and blotted to nitrocellulose (Bio-Rad). The membrane was incubated in 3% BSA as a blocking agent followed by primary anti-GFP rabbit specific antibody, 1:1000 dilution; followed by secondary antibody, anti-rabbit AP antibody produced in goat (Sigma), 1:5000 dilution. The immunosignal was revealed by NBT/BCIP staining.

### Accession Numbers

MtSyt1: Medtr4g073400. Sequence ID: XM_003607150.1.
MtSyt2: Medtr1g025550. Sequence ID: XM_003589467.1.
MtSyt3: Medtr1g094810. Sequence ID: XM_003591831.1.
MtExo 70i: Medtr1g017910. Sequence ID: XM_003589006.1.

## Results

### The Selection of *Medicago truncatula* Homologs of Synaptotagmin1

Five homologs of synaptotagmin1 genes were retrieved from *M. truncatula* databases by BLAST analysis using *Arabidopsis thaliana* synaptotagmin1 sequence. Phylogenetic analysis shows that the *MtSyt1* and *MtSyt2* genes and *A. thaliana AtSYT1, AtSYT2* belong to the same group, and annotated in NCBI as synaptotagmins7 of *M. truncatula*, further sub-grouping is not clearly defined (Supplementary Figure 1). *MtSyt1* is a closest homolog of *AtSYT1. MtSyt3* is an ortholog of *AtSyt3*, forming a separate group (Yamazaki et al., 2010). *MtSyt4* and *MtSyt5* are grouped with *AtSyt4* and *AtSyt5.*

For this study we selected *MtSyt1, MtSyt2*, and *MtSyt3* genes. The expression of *MtSyt1, MtSyt2*, and *MtSyt3* in roots and nodules at 14 dpi was analyzed by qRT-PCR (Supplementary Figure 2). *MtSyt1* and *MtSyt2* were expressed less in nodules than in the roots, whereas *MtSyt3* expression in nodules was higher. According to Symbimix database (Roux et al., 2014; INRA^3^) *MtSyt1*, MtSyt2 and *MtSyt3* are expressed in the meristem, zone of infection and zone of nitrogen fixation; the expression of *MtSyt1* and *MtSyt3* is higher than one of *MtSyt2* (Supplementary Figure 2B). To clarify the role of synaptotagmins during symbiosis development, we studied the expression, localization, and functional role of *MtSyt1, MtSyt2* and *MtSyt3*.

### Expression Analyses of *MtSyt1, MtSyt2*, and *MtSyt3*

**FIGURE 1:**
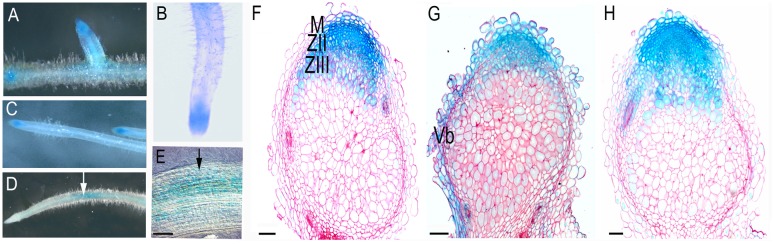
**Promoter-GUS expression analysis of *ProMtSyt1:GUS, ProMtSyt2:GUS*, and *ProMtSyt3:GUS* in roots and nodules. (A)** Expression pattern of *ProMtSyt1:GUS;*, **(B)** *ProMtSyt2:GUS;***(C)** *ProMtSyt3:GUS* in roots. Synaptotagmins have a low ubiquitous expression in all cells, GUS staining is most intense in the meristems and vascular bundles. **(D)** *ProMtSyt1: GUS* in the root after bending. **(E)** The magnification of **(D)** shows the topography of GUS staining. The bending (arrow) is causing the repositioning of the GUS expression to the place of curvature. Expression pattern of *ProMtSyt1:GUS* **(F)**, *ProMtSyt2:GUS* **(G)** and *ProMtSyt3:GUS* **(H)** in nodules, show the strong expression in the nodule meristem, vascular bundles, zone of infection and proximal layers of the fixation zone. M, meristem; ZII, zone of infection; ZIII, zone of fixation; Vb, vascular bundle. Bars: **(E–H)** = 100 μm.

For the analysis, we created constructs containing a region 2.5 kb upstream of the translational start of *MtSyt1, MtSyt2* and *MtSyt3* fused to GUS. Transgenic roots were obtained using *Agrobacterium rhizogenes*-mediated transformation. In the roots *ProMtSyt1:GUS, ProMtSyt2:GUS* and *ProMtSyt:GUS* were expressed in all cells and most strongly in root primordia, meristem, epidermis of the zone of elongation, including the root hairs (**Figures 1A–B**). To see whether the expression pattern may be affected by membrane tension, we performed a root bending assay. Transgenic roots were selected from the plants grown in vertically oriented agar plates, bent and fixed using the tooth sticks. Bent roots were harvested after 48 h. Bending the root shifted the GUS signal to the site of curvature (**Figures 1D,E**). We concluded that the expression of synaptotagmins follows the region of the root with high membrane tension caused by the bending, hence the created constructs: *ProMtSyt1:GUS, ProMtSyt2:GUS*, and *ProMtSyt3:GUS* give a functional response for mechanical stimulation. In the nodules, *ProMtSyt1:GUS, ProMtSyt2:GUS*, and *ProMtSyt3:GUS* were strongly expressed in cells undergoing rapid increase of volume: in the cells of the meristem, of distal cell layers of the infection zone (**Figures 1F–H**) and distal layers of the zone of fixation (**Figures 1F,H**) (*ProMtSyt1:GUS* and *ProMtSyt3:GUS).*

### Cellular Localization of MtSyt1, MtSyt2, and MtSyt3

**FIGURE 2:**
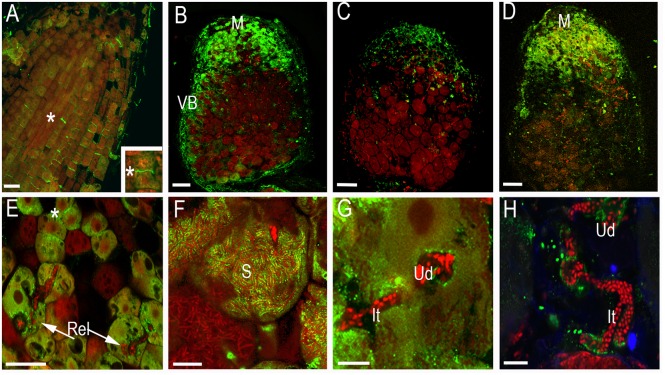
**The localization of GFP-tagged MtSyt1, MtSyt2 and MtSyt3 by confocal microscopy immunolabeling with anti-GFP antibody.** In root meristem the synaptotagmin signal is quite intense over developing cell walls in freshly divided meristematic cells (^∗^) (ProMtSyt2:MtSyt2-GFP) **(A)**. The localization of synaptotagmins in apical part of root nodules: ProMtSyt1:MtSyt1-GFP **(B)**, ProMtSyt2:MtSyt2-GFP **(C)**, ProMtSyt3:MtSyt3-GFP **(D)**. On the cellular level the signal of synaptotagmins is associated with newly formed cell walls in nodule meristem, with infection threads/unwalled droplets on the place of bacteria release. ProMtSyt1:MtSyt1-GFP **(E)**; symbiosomes labeled by ProMtSyt1:MtSyt1-GFP **(F)**. ProMtSyt2:MtSyt2-GFP **(G)**, ProMtSyt3:MtSyt3-GFP **(H)**. Vb, vascular bundle; Rel, release of bacteria; It, infection thread; Ud, unwalled droplet; M, meristem; S, symbiosomes; PM, plasma membrane; asterisk(^∗^) developing cell wall. Bars: **(A–D)** = 100 μm, **(E)** = 25 μm, **(H,G)** = 5 μm, **(H)** = 10 μm. Color codes: synaptotagmins: green fluorescence, rhizobia and nuclei of host cell: red fluorescence. Single optical sections.

**FIGURE 3:**
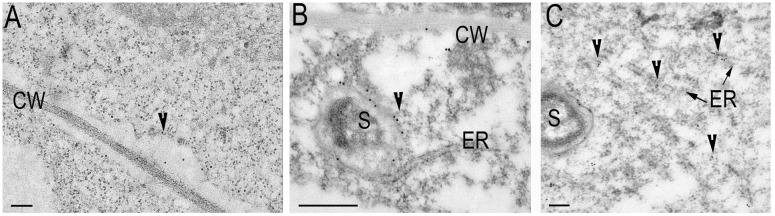
**Electron microscopy (EM) immunogold labeling of Syt1 (A–C)**: The signal over the plasma membrane (PM) of freshly divided cells **(A)**; the labeling on symbiosome membrane, note the contact with ER **(B)**; **(C)** The signal on symbiosome membrane and ER. Gold granules are pointed by arrowheads. CW, cell wall; ER, endoplasmic reticulum; S, symbiosome. Bars: **(A)** = 200 nm, **(B)** = 500 nm, **(C)** = 200 nm

To investigate the localization of synaptotagmins in nodules and roots, constructs expressing GFP translational fusions at the C-terminal position of these genes under the control of their respective 2.5 kb native 5′ regulatory sequences were created. The size of the proteins has been analyzed by Western blot with anti-GFP antibody (Supplementary Figure 3). For confocal imaging, roots and root nodules at 14–21 dpi were hand sectioned and fixed in 1% paraformaldehyde in a phosphate buffer as previously described (Gavrin et al., 2014). The GFP signal was weak and has to be enhanced by anti-GFP antibody coupled with secondary antibody tagged by ALEXA488. Confocal microscopy of the tissue reveals that GFP-tagged proteins ProMtSyt1:MtSyt1-GFP, ProMtSyt2:MtSyt2-GFP and ProMtSyt3:MtSyt3-GFP locally accumulate in the meristem of roots, the strongest signal was found in the developing cell walls in freshly divided cells (**Figure 2A**). In the nodules, the immunosignal of ProMtSyt1:MtSyt1-GFP, ProMtSyt2:MtSyt2-GFP and ProMtSyt3:MtSyt3-GFP was present in apical part of the nodule: in the meristem, zone of infection and proximal cell layers of zone of fixation and in vascular bundles (**Figures 2B–D**). On the cellular level the signal was found in the ER-rich central areas of the cell with an enrichment of the signal over the PM of freshly divided meristematic cells and around infection threads and unwalled droplets (**Figures 2E,G,H**). The symbiosomes were specifically outlined by the GFP signal only in the nodules carrying ProMtSyt1:MtSyt1-GFP (**Figure 2F**). The nodule tissue from the negative control where the primary (anti-GFP) antibody was omitted during the labeling process does not display labeling pattern (Supplementary Figure 4A). Synaptotagmins were therefore localized in the cells with expanding membranes. The special role in symbiosome membrane expansion, according to the localization study, belongs to MtSyt1 which is localizes on symbiosome membrane. Electron microscopy (EM) immunogold analysis of the transgenic nodules carrying GFP-tagged synaptotagmin shows the signal over the PM of freshly divided cells, in ER and the symbiosome membrane in the transgenic nodules carrying ProMtSyt1:MtSyt1-GFP (**Figures 3A–C**). The control without primary (anti-GFP) antibody is shown on Supplementary Figure 4B.

Endoplasmic reticulum is extremely abundant in young, freshly infected cells. ER contact sites with the membranes of infection threads, unwalled droplets, and symbiosomes represented as local dilations of tubular extensions and vesicles are ubiquitously present in young infected cells. The images obtained by transmission EM show numerous fusion events with the membrane vesicles and close contacts with ER (Supplementary Figures 5A,B).

To check the functional status of GFP-tagged constructs, we compared the localization pattern of the construct with GFP at the C-terminal position against the construct containing N-terminal (transmembrane domain) GFP (ProMtSyt3-GFP: MtSyt3). The GFP positioning on the transmembrane domain resulted in the protein becoming mis-localized. According to the localization pattern, the GFP signal was not targeted to the PM and was retained in the cytoplasm forming a dot-like pattern (Supplementary Figure 6A). EM immunogold analysis has shown that the protein was mainly present over the ER and in Golgi bodies (Supplementary Figure 6B). Therefore the C-terminal constructs which were used for the localization study maintained the correct positioning in the PM of the host cell.

### Functional Analysis Using Double Silencing of *MtSyt1/MtSyt3* and *MtSyt2/MtSyt3*

We believe that the null mutations in case of housekeeping genes like synaptotagmins negatively affect the development of the whole host plant and indiscriminately damage the nodules. Hereafter, to specify the role of synaptotagmins in the infected symbiotic cells and avoid the negative effects for the shoots, vascular system and non-infected nodule cells we used RNAi silencing under the control of *Enod12* promoter. This promoter is active in the nodules in zone of infection (Limpens et al., 2005), this approach permits to silence genes only in the young infected cells without affecting the other cells of the nodule.

We have created the double silencing constructs of *MtSyt1/MtSyt3* and *MtSyt2/MtSyt3* to induce a simultaneous silencing under the *Enod12* promoter. The level of silencing of *MtSyt1/MtSyt3* and *MtSyt2/MtSyt3* in transgenic nodules of *ProENOD12:MtSyt1/MtSyt3* and *ProENOD12:MtSyt2/MtSyt3* is shown in Supplementary Figure 7.

**FIGURE 4:**
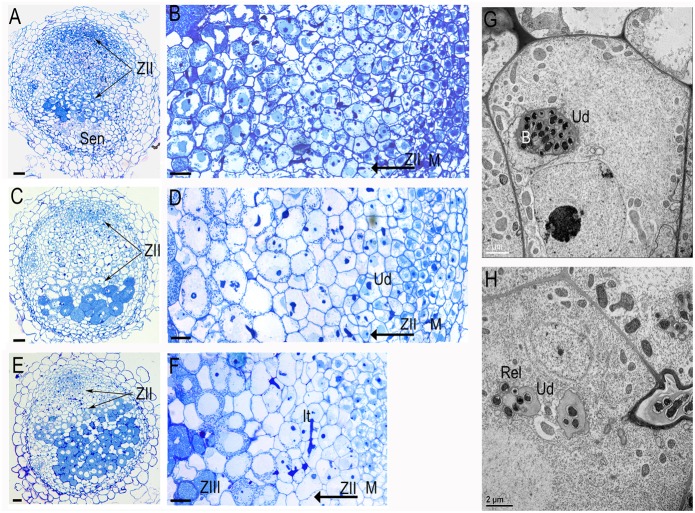
**Functional analysis of synaptotagmins using double silencing constructs *ProENOD12:MtSyt1/MtSyt3* and *ProENOD12:MtSyt2/MtSyt3* analyzed by light (A–F)** and electron microscopy **(G,H)**. Nodules elicited on the transgenic roots: *ProENOD12:MtSyt1/MtSyt3* **(A,B)**, *ProENOD12:MtSyt2/MtSyt3* **(C,D)**. Note the extended zone of infection (zoneII) (arrows) and the senescent cells on the basal part of the nodules. **(B)** The magnification of **(A,D)** the magnification of **(C)** showing the extended zone of infection, note the small immature bacteroides populating the infected cells of zoneII. **(E,F)** Control nodule. Note the short zone of infection followed by zone of nitrogen fixation (zoneIII) containing mature infected cells with large developed bacteroides and the starch granules. **(G)** EM image displays the cells containing the unwalled droplet without the bacteria release from the transgenic nodules of *ProENOD12:MtSyt1/MtSyt3*, **(H)** control nodules of the same stage of development as **(G)** with unwalled droplets and releasing rhizobia. ZII, zone of infection; ZIII, zone of nitrogen fixation; Sen, zone of senescence; It, infection thread; Ud, unwalled droplet; B, bacteria; Rel, release of rhizobia. Bars: **(A,C,E)** = 50 μm; **(B,D,F)** = 25 μm, **(G,H)**: as indicated.

Analysis of the transgenic roots from 10 plants showed that transgenic nodules were smaller and less numerous: 2.81 ± 1.65 per plant versus 5.32 ± 1.91 in the controls. Transgenic nodules shown a distinct phenotype: a short meristem, extended zone of infection and diminished or aborted zone of fixation (**Figures 4A–D** versus **Figures 4E,F**). Zones of infection have increased numbers of cell layers (**Table 1**), delayed rhizobia release and symbiosome maturation. The double-silencing phenotype was observed in 65% of the nodules (*n* = 20). The analysis was performed on 20 nodules randomly collected from 10 plants. In the majority of affected nodules, the symbiosomes were not able to differentiate beyond stage 2/3 according to the classification of Vasse et al. (1990) and undergo premature senescence (**Figure 4A**). EM analysis of nodules with double silencing (*ProENOD12:MtSyt1/MtSyt3* and *ProENOD12:MtSyt2/MtSyt3)* shown that infected cells in the extended zone of infection contain unwalled droplets without apparent bacteria release (**Figures 4G,H**). However, the release was not completely inhibited and the cells were finally colonized, but the maturation of the symbiosomes was restricted. The colonization of infected cells was quite slow and was taking up to 12 cell layers (cf. **Figures 4A,B** with **Figure 4E**) comparing with 3–5 in control. The number of cell layers in different nodule zones in control and RNAi nodules and the statistical analysis are presented in **Table 1**. The *T*-test was used to estimate a difference between control and RNAi nodules (*P* < 0.05).We have concluded that the double silencing of *MtSyt1/MtSyt3* and *MtSyt2/MtSyt3* negatively affects nodule meristem development, growth of infected cells, caused the reduction of bacteria release from unwalled droplets, and hampers growth and maturation of symbiosomes.

**Table 1:** The number of cell layers in different nodule zones in control and RNAi nodules.

	Meristem	Infection zone and interzone II/III	Zone of nitrogen fixation
Control nodules	3.5 ± 0.23	5.75 ± 0.44	21.3 ± 2.74
RNAi nodules	0.9 ± 0.16	11.30 ± 1.65	1.84 ± 1.6

*The difference between control and RNAi nodules is significant (P < 0.05, T-test).*

### The Localization of *MtSyt1, MtSyt3* in Arbusculated Root Cells in Comparison with Exo70i

To specify the role of synaptotagmins in the formation of interface membrane in other type of symbiosis we have studied the localization of MtSyt3 in the roots inoculated by *R. intraradices.* The formation of arbuscules, the symbiotic intracellular extensions of hyphae is always accompanied by the rapid increase of interface membrane (Genre et al., 2009; Gutjahr and Parniske, 2013). The distribution of GFP signal of ProMtSyt1:MtSyt1-GFP and ProMtSyt3:MtSyt3-GFP was similar, it was clearly delineating the membranes of arbuscules fine branches, but not the trunks (Supplementary Figures 8A,B). The GFP signal was enhanced by anti-GFP antibody coupled with secondary antibody tagged by ALEXA488 equally as it was done for the nodule tissue.

To specify the role of synaptotagmin in tip growth we have compared the localization of synaptotagmin with the localization of Exo70i, the subunit of tethering complex exocyst in root nodule infected cells and in the cells containing arbuscules. For the study we have cloned the *Exo70* subunit which is highly expressed in arbusculated roots and in the nodules. The same gene have been cloned and characterized by Zhang et al. (2015), due to this we are using the abbreviation used by Zhang et al. (2015): *Exo70i*, despite that this gene was cloned by us independently.

In arbusculated root cells Exo70i was strictly localized near the tip of fine branches of arbuscules in contrast to the signal of synaptotagmin which was found over the whole surface of the branches (Supplementary Figure 8C versus Supplementary Figures 8A,B). The localization pattern of this subunit over the arbuscules in our work and in the paper of Zhang et al. (2015) shows the identical pattern.

The localization of Exo70i in infected cells of the nodules was specific, it has dot-like pattern and forms a small clusters near the tips of infection threads and also was marking unwalled droplets, but was not delineating the membrane of infection thread or unwalled droplet like synaptotagmins do, neither it was labeling the symbiosome membrane (Supplementary Figure 8D).

Therefore we concluded that the distribution of synaptotagmins on the membranes of infection threads and fine branches of arbuscules was spatially overlapping on the regions of infection threads tips and the tips of arbuscular fine branches, but was not overlaying with the signal of Exo70i on the whole interface membrane.

## Discussion

The intracellular accommodation of rhizobia triggers the reformation of endomembrane system, redirection of vesicular traffic and reorganization of the cytoskeleton and vacuole of a host cell (Roth and Stacey, 1989; Parniske, 2000; Gibson et al., 2008; Ivanov et al., 2012; Kondorosi et al., 2013; Gavrin et al., 2014, 2015, 2016; Gavrin, 2015). As a result of these events the symbiotic interface membrane is formed. We hypothesized that the membrane tension created by expanding microsymbiont provides the vector for targeted endomembrane traffic toward the symbiotic interface. To address this hypothesis, we performed promoter-GUS analyses, the localization study, and functional analysis of synaptotagmins *MtSyt1, MtSyt2* and *MtSyt3.* As it was expected synaptotagmins, involved in housekeeping functions of membrane fusion, gave markedly enhanced signal over the “symbiotic” unsymmetrical protrusions of PM in zone of infection. We believe that the local accumulation of synaptotagmins over the interface enveloping expanding microbes represents functional membrane subcompartmentalization (Jarsch et al., 2014; Konrad and Ott, 2015) and may be causal for enhanced fusion capacity of these subcompartments. Synaptotagmins in this situation are serving as a “beacons” for a vectorial membrane transport. We do not consider it to be a “symbiotic” function, but rather a house keeping function of the host cell used for urgent membrane proliferation. For example, the localization pattern of *Nicotiana benthamiana* synaptotagmin homolog on the membrane enveloping the haustorium formed by *Phytophthora infestans* was quite similar to the pattern observed in our experiments with infection threads and arbuscular thin branches (Lu et al., 2012; Bozkurt et al., 2014).

The localization of MtSyt1 on symbiosome membrane is especially interesting. Symbiosomes are detached from PM, however, the surrounding symbiosome membrane surface reach up to several folds of PM of host cell (Roth and Stacey, 1989). The complete maturation of symbiosomes takes 5–7 cell layers, with most rapid growth in 1–2 cell layers proximal to zone of nitrogen fixation (Gavrin et al., 2014). This speedy growth brings under the consideration the putative membrane resources for the symbiosome membrane. The most obvious source is an exocytotic pathway with post-Golgi vesicles (Van de Velde et al., 2010; Wang et al., 2010; Ivanov et al., 2012; Sinharoy et al., 2016). However, since long ER has been considered to be one of the sources of membrane for symbiosomes (Roth and Stacey, 1989; Clarke et al., 2015), it is extremely abundant in young infected cells (Ivanov et al., 2012). Recently it was reported that ER is the main membrane source for biogenesis of the lytic vacuole in *Arabidopsis* meristem Viotti et al. (2013). As an organelle ER consists of discrete functional domains, it is quite dynamic and change a tubular to a vesicular morphology (Sparkes et al., 2009; Raeymaekers and Larivière, 2011). We speculate that the synaptotagmins may create a “reaper points” on symbiosome membrane for the fusion with ER. The putative SNARE complexes in infected cells may include the homologs of SNARE *NPSN11*, SNARE *SYP71*, and *VAMP721, 722* which are forming tetrameric SNARE complex on the cell plates during cytokinesis in *Arabidopsis* (El Kasmi et al., 2013). In the previous work we have found that v-SNAREs *MtVAMP721d* and *MtVAMP721a* are essential for the intracellular accommodation of microsymbionts in nodules and arbusculated roots of *M. truncatula*. These SNAREs localize on symbiosome membrane and on the membranes of fine branches of arbuscules, as well as on the cell plate (Ivanov et al., 2012).

The comparison of localization pattern of *MtSyt1, MtSyt2*, and *MtSyt3* showed also some unexpected differences. The localization on the symbiosome membrane is a result of retargeting from the default targeting to host cell PM. The retargeting toward symbiotic interface depends on the level of expression (Pumplin et al., 2012) as well as on other factors including the reformation of actin network around the symbiosomes (Gavrin et al., 2015). However, *MtSyt3*, the homolog of *AtSyt3*, which has similar expression pattern with *MtSyt1* and localizes in apical part of the nodule and in proximal cell layers of zone of nitrogen fixation was not localized on the symbiosome membrane in contrast to *MtSyt1*. The functional features of Syt3 in plants are not defined, however, for the explanation we can point to the features of animal cells Syt3 (SytIII). Apart of being on PM (Bhalla et al., 2008) it colocalizes with early endosomal markers (Grimberg et al., 2003). In previous work we have found that symbiosomes do not accept early endosome markers (Limpens et al., 2009), so it may be one of the reasons why MtSyt3 is not retargeted toward symbiosome membrane.

The functional analysis of *MtSyt1, MtSyt2*, and *MtSyt3* using double RNAi of *MtSyt1/MtSyt2* and *MtSyt2/MtSyt3* confirms the role of synaptotagmins in formation of symbiotic membrane interface and its growth. The silencing causes a delay in bacteria release and maturation and consequent widening of the infection zone. Such type of the deviations in symbiosomes development reflects the defect in colonization by rhizobia (Sinharoy et al., 2016). This phenotype resembles the phenotype of nodules with silencing of vesicle-associated membrane protein VAMP72d/a, the key player in targeted membrane fusion to PM and to symbiotic interface in nodules and micorriza (Ivanov et al., 2012). The restriction of symbiosomes development to stage 1–2 was also observed in the mutant *dnf1* (Wang et al., 2010). *Dnf1* encodes a symbiosis-specific subunit of the signal-peptidase complex (Van de Velde et al., 2010; Wang et al., 2010), a component of the protein secretory pathway in the ER.

With the aim of determining the role of synaptotagmins in interface membrane formation, we compared the localization of MtSyt1, MtSyt3 and exocyst subunit EXO70i in root nodules and arbusculated roots. The localization patterns of MtSyt1, MtSyt3 and EXO70i has shown that subcompartments labeled by synaptotagmins are broader and include lateral parts of infection threads and the membranes of unwalled droplets, which are the structures with an isodiametric type of growth, MtSyt1 also labels symbiosomes. This suggests that not only tip-targeted post-Golgi vesicles but also other membrane resources like, for instance, ER may be involved in the growth of interface membrane.

## Conclusion

The synaptotagmin-dependent membrane fusion activated by the change in the membrane tension around the expanding microsymbionts along with tip-targeted exocytosis is operational in the formation of symbiotic interface.

## Author Contributions

EF and AG designed the research. AG, EF, and OK performed experiments and analyzed data. AG and EF wrote the manuscript. EF, AG, OK, and TB edited the manuscript.

## Conflict of Interest Statement

The authors declare that the research was conducted in the absence of any commercial or financial relationships that could be construed as a potential conflict of interest.

## Footnotes

1 http://mtgea.noble.org/v2/

2 https://iant.toulouse.inra.fr/symbimics/

3 https://iant.toulouse.inra.fr/symbimics/
