# Supplementary material for: Interface Symbiotic Membrane Formation in Root Nodules of Medicago truncatula: the Role of Synaptotagmins MtSyt1, MtSyt2 and MtSyt3
Source: Front Plant Sci. 2017 Feb 20;8:201. doi: 10.3389/fpls.2017.00201 (PMC5316549; doi:10.3389/fpls.2017.00201)
Supplement: Supplementary file 1 [file Data_Sheet_1.PDF]

Supplementary materials

**Supplementary Figure 1.** The phylogenetic tree of *M. truncatula* synaptotagmin orthologs *MtSyt1*, *MtSyt2* and *MtSyt3* grouped with *A. thaliana* synaptotagmins (AtSYTs).

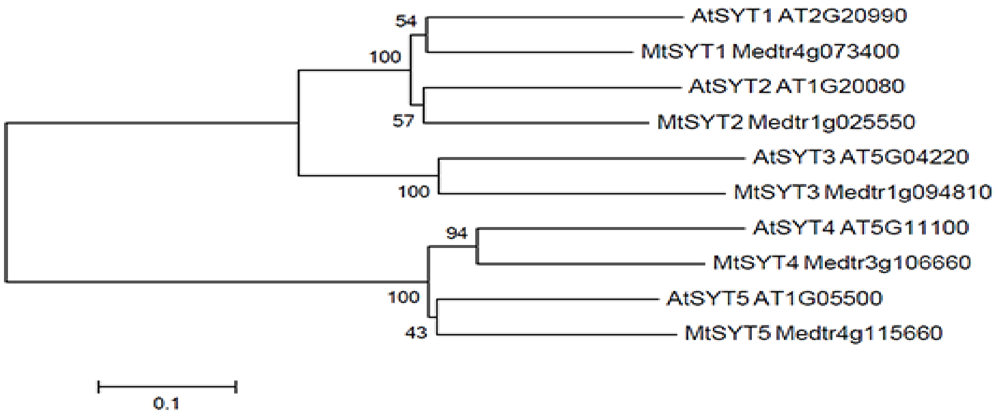


**Supplementary Figure 2 A,B.** The expression level of *MtSyt1*, *MtSyt2*, *MtSyt3* in roots and 14 dpi nodules (A). The expression (deseq-normalized RNA-seq reads) of *MtSyt1*, *MtSyt2*, *MtSyt3* in roots and nodules according to Symbimix database (Roux et al., 2014) https://iant.toulouse.inra.fr/symbimics/ (B).

A

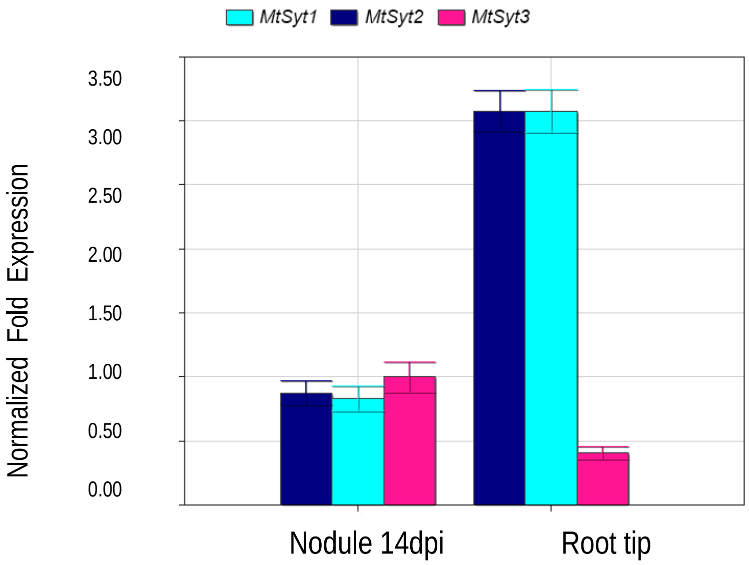


Supplementary Figure 2A. The expression level of *MtSyt1*, *MtSyt2*, *MtSyt3* in roots and nodules. Error bars represent standard deviation. The difference between the expression of *MtSyt3* in roots and nodules is significant (*P*<0.05, T-test).

B

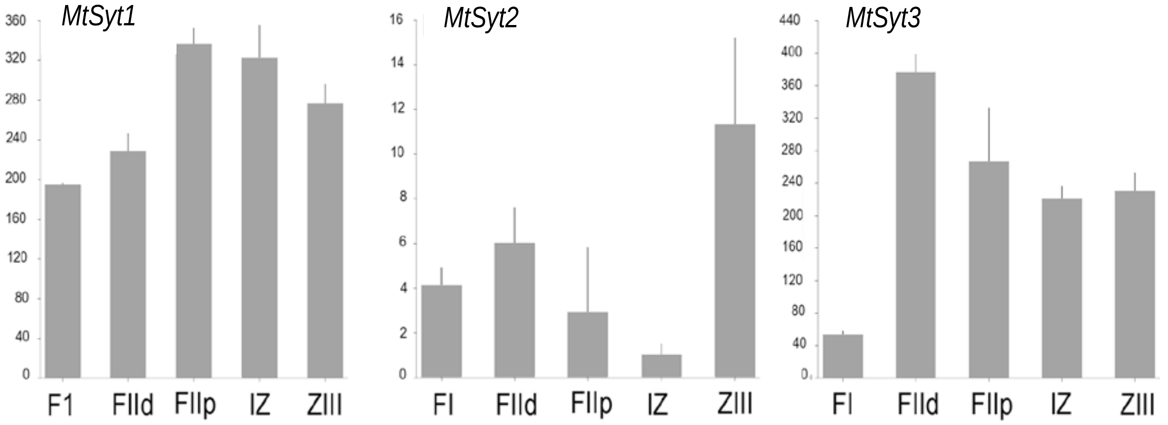


Supplementary Figure 2B. The expression (deseq-normalized RNA-seq reads) of *MtSyt1*, *MtSyt2*, *MtSyt3* in roots and nodules. The distribution of normalized RNA-seq reads (%) for *MtSyt1*, *MtSyt2*, *MtSyt3* in nodule's developmental zones: FI, meristematic zone according to Symbimix database. Abbreviations: FIId, distal infection zone; FIIp, proximal infection zone; IZ, interzone II/III; ZIII, fixation zone.

Supplementary Figure 3. Western blot with anti-GFP antibody

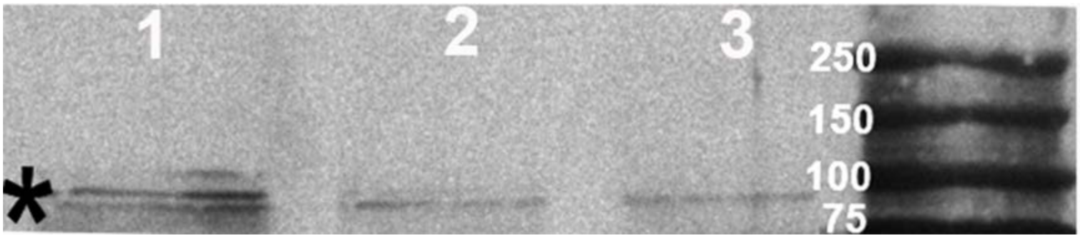


Supplementary Figure 3. Crude extracts from the transgenic nodules: line1: ProMtSyt1:MtSyt1-GFP (band =88.38kD), line (2): ProMtSyt2:MtSyt2-GFP (band=87.94kD), line 3: ProMtSyt3:MtSyt3-GFP (band=87.14kD). The membrane is treated by anti-GFP antibody, ( *) the band.

Supplementary Figure 4. The negative control for immunolabelling where the primary (anti-GFP) antibody was omitted during the labelling.

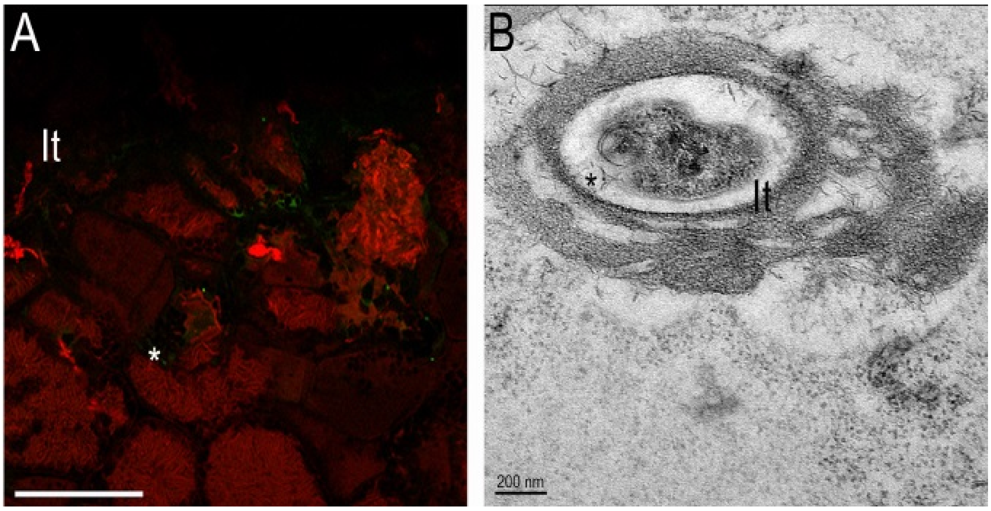


Supplementary Figure 4. The primary (anti-GFP) antibody was omitted (A): confocal microscopy, note absence of green fluorescence pattern in apical part on the nodule, only few background fluorophore dots, bar: (A)50 μm; (B) immunogold control, infection thread, background gold particle (*), bar: as indicated

Supplementary Figure 5(A,B). EM analysis of the distribution of endoplasmic reticulum (ER) in infected cells

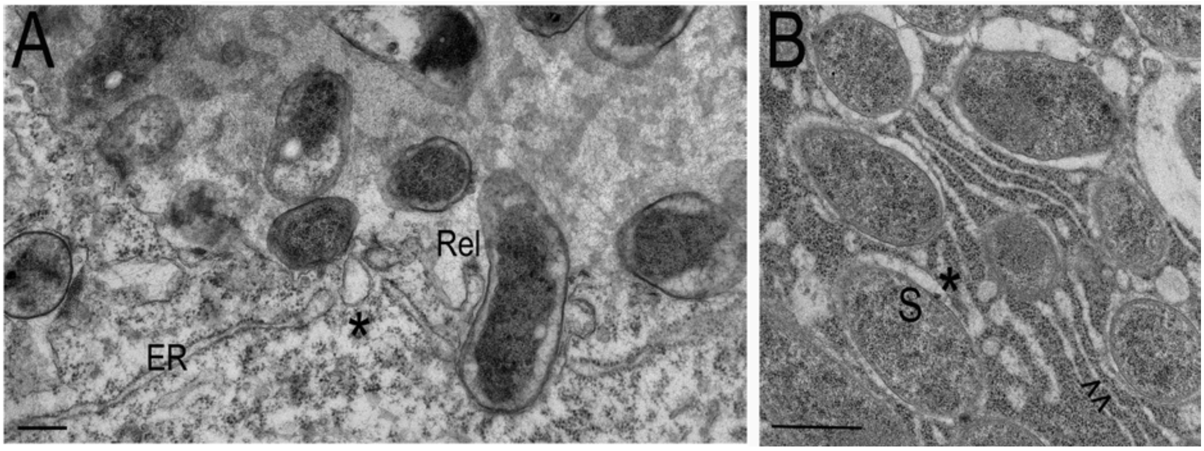


(A) Membrane contacts of ER with interface membrane in the site of bacteria release, and (B) with young symbiosomes. CW:cell wall, ER:endoplasmic reticulum, S:symbiosome, Rel:release of rhizobia from unwalled droplet, B: bacterium, It-infection thread, (*) dilation of ER, (^^) - the merging membranes. Bars: (A),(B)=500nm.

Supplementary Figure 6 A,B. The localization of MtSyt3 in the transgenic nodules carrying the construct ProMtSyt3-GFP:MtSyt3

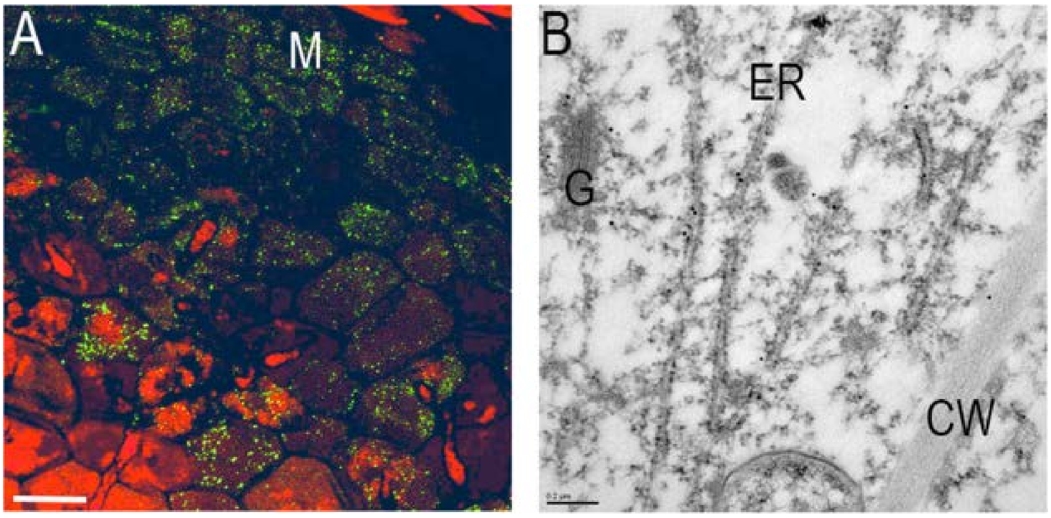


Supplementary Figure 6A,B. (A)Confocal microscopy, the dot-like immunosignal is present in the cytoplasm of the cells in meristem and zone of infection. (B) EM immunogold analysis shows the gold labelling over the ER and Golgi. Bars:(A):25μm, (B):200nm. M:meristem, ZII:zone of infection, ER:endoplasmic reticulum, CW:cell wall, G:Golgi apparatus

Supplementary Figure 7. The level of silencing for *MtSyt1*, *MtSyt2* and *MtSyt3* estimated by q-PCR analysis on the templates from transgenic nodules with double silencing: *ProENOD12:MtSyt1/MtSyt3* and *ProENOD12:MtSyt2/MtSyt3.*

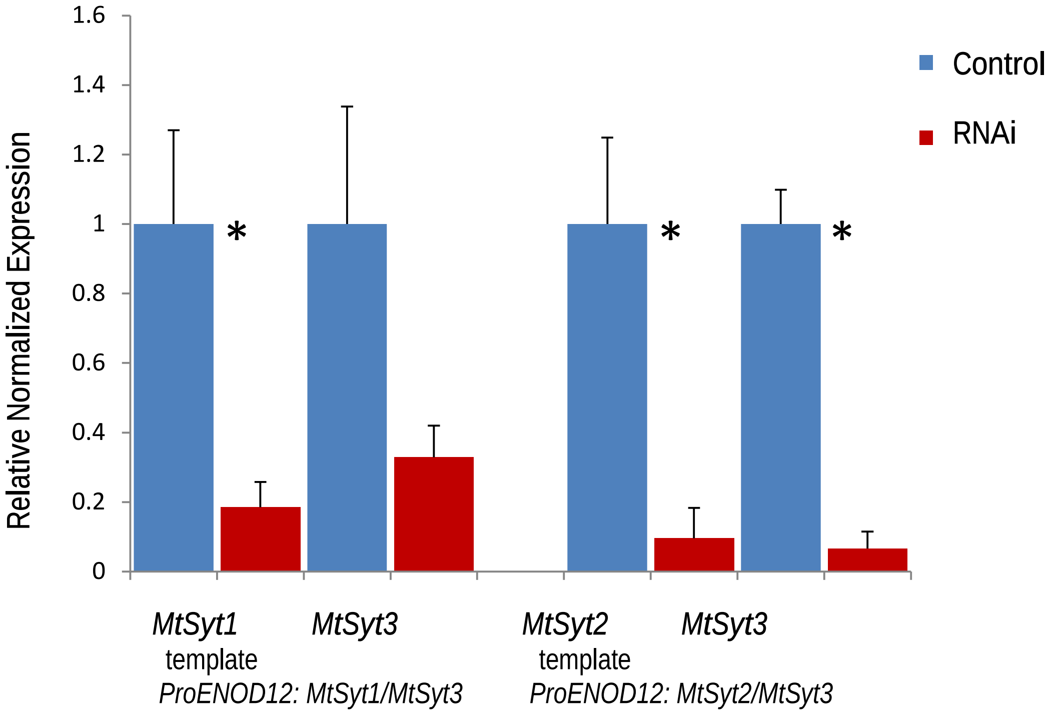


Significant difference (*), T-test.

Supplementary Figure 8. The localization of ProMtSyt1:MtSyt1-GFP, ProMtSyt3:MtSyt3-GFP in arbusculated root cell , the localization of ProUbiq3:GFP-Exo70i in arbusculated cells and in root nodule (GFP signal enhanced with anti-GFP Ab.

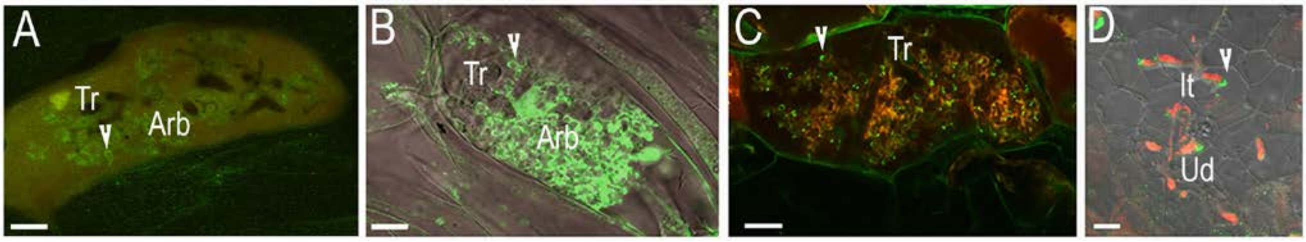


Supplementary Figure 8 (A,B,C,D). (A), Immunosignal of over the membrane of the fine branch of arbuscule, MtSyt1(A), GFP signal without antibody enhancement; (B), MtSyt3, GFP signal enhanced with anti-GFP Ab;.(C), The localization of Exo70i (ProUbiq3:GFP-Exo70i) in arbusculated root cell, arrowhead: note the signal over the tips of fine branches, (D): Exo70i in root nodule, arrowhead: the signal is situated near the tips of infection threads and also marked some patches on unwalled droplets (D). Arrowhead: note strong immunosignal over the membrane of the fine branch of arbuscule. arrowhead: note the signal over the tips of fine branches, Exo70i in root nodule, arrowhead: the signal is situated near the tips of infection threads and also marked some patches on unwalled droplets. It:infection thread, Arb-arbuscule, Tr-trunk of arbuscule. (A),(B),(C),(D)=10µm. Color codes: synaptotagmins: green fluorescence, rhizobia and nuclei of host cell: red fluorescence. Single optical sections.

Supplementary Table 1. List of primers

Cloning primers

| | |
|---|---|
| MtSyt1-ORF-F | ATGGGTTTCTTCAGTACAATTTTTGG |
| MtSyt1-ORF-R | TTATGCAGTTCTCCACTGCAAC |
| MtSyt2-ORF-F | ATGAGTATTTTAAGTACTATAGCTAGTTTTTTAGG |
| MtSyt2-ORF-R | TTATGGGGTTCTCCACTGAA |
| MtSyt3-ORF-F | ATGGGGTTCTTTGAAAGTTTCTT |
| MtSyt3-ORF-R | TTAAACCACCTTCCATTTTATCTCA |
| MtSyt1-Pr-1-F | AGAATCCCTTATTGTACTTTTTGC |
| MtSyt1-Pr-1-R | TTGGAATGGATCTAAATGATTTC |
| MtSyt2-Pr-2-F | CGACGTTCCTTCCTCTTTGG |
| MtSyt2-Pr-2-R | TTCAACTTCAATATCAATGTCTATGG |
| MtSyt3-Pr-3-F | AGCAACTAAAAATGGCAAGAAAA |
| MtSyt3-Pr-3-R | TTTTGGTGAACAGAGCAATGA |
| Exo70i -ORF-F | ATGGCATTGTTAATGGTTCCTC |
| Exo70i-ORF-R | TCTAGACCTTCTGATATCAGTTTCCA |

qPCR primers

| | |
|---|---|
| MtSyt1-F | AGGAGGCCCGTTGGAATTTT |
| MtSyt1-R | TGTGATGCTTCCCCTCAACA |
| MtSyt2-F | TGGATCCATCACAGGCCATG |
| MtSyt2-R | CGCAAACGGATTTGTGTGGT |
| MtSyt3-F | TTCCGGCGAAGAAAACCACT |
| MtSyt3-R | ATGCCCCAGGGATTCCTTTG |

Primers for double silencing

| | |
|---|---|
| MtSyt1-F | TGGATCCATCACAGGCCATG |
| Mt Syt1-R | CGCAAACGGATTTGTGTGGT |
| MtSyt2-F | AGGAGGCCCGTTGGAATTTT |
| MtSyt2-R | TGTGATGCTTCCCCTCAACA |
| MtSyt3-F | TTCCGGCGAAGAAAACCACT |
| MtSyt3-R | ATGCCCCAGGGATTCCTTTG |
